# Continuous feeding strategy for polyhydroxyalkanoate production from solid waste animal fat at laboratory‐ and pilot‐scale

**DOI:** 10.1111/1751-7915.14104

**Published:** 2022-08-03

**Authors:** Björn Gutschmann, Matilde Maldonado Simões, Thomas Schiewe, Edith S. Schröter, Marvin Münzberg, Peter Neubauer, Anika Bockisch, Sebastian L. Riedel

**Affiliations:** ^1^ Technische Universität Berlin Chair of Bioprocess Engineering Berlin Germany; ^2^ innoFSPEC University of Potsdam Potsdam Germany; ^3^ Bio‐PAT e.V Berlin Germany

## Abstract

Bioconversion of waste animal fat (WAF) to polyhydroxyalkanoates (PHAs) is an approach to lower the production costs of these plastic alternatives. However, the solid nature of WAF requires a tailor‐made process development. In this study, a double‐jacket feeding system was built to thermally liquefy the WAF to employ a continuous feeding strategy. During laboratory‐scale cultivations with *Ralstonia eutropha* Re2058/pCB113, 70% more PHA (45 g_PHA_ L^−1^) and a 75% higher space–time yield (0.63 g_PHA_ L^−1^ h^−1^) were achieved compared to previously reported fermentations with solid WAF. During the development process, growth and PHA formation were monitored in real‐time by *in‐line* photon density wave spectroscopy. The process robustness was further evaluated during scale‐down fermentations employing an oscillating aeration, which did not alter the PHA yield although cells encountered periods of oxygen limitation. Flow cytometry with propidium iodide staining showed that more than two‐thirds of the cells were viable at the end of the cultivation and viability was even little higher in the scale‐down cultivations. Application of this feeding system at 150‐L pilot‐scale cultivation yielded in 31.5 g_PHA_ L^−1^, which is a promising result for the further scale‐up to industrial scale.

## INTRODUCTION

Plastics play an important role in all areas of our daily lives. In 2019 alone, 368 million tons of plastics were produced globally (PlasticsEurope, 2020). There is no doubt that plastic production must shift from a fossil‐based to a renewable resource‐based production to reduce greenhouse gas emissions. As plastics also play a major role in polluting the ecosystem, the development of bio‐based and biodegradable alternatives is desirable. Polyhydroxyalkanoates (PHAs) are a class of natural polyesters that possess those two attributes (Riedel & Brigham, 2020).

Polyhydroxyalkanoates are synthesized inside the cytoplasm of bacterial cells as carbon and energy storage compounds. They can be produced by industrial fermentation from various renewable resources and biogenic waste streams (Andreasi Bassi et al., 2021; Bhatia et al., 2021; Riedel & Brigham, 2019, 2020; Taguchi & Matsumoto, 2021; Wang et al., 2021). Oleaginous feedstocks are particularly attractive for PHA production because of their high carbon contents, high conversion rates, and lower culture dilution in fed‐batch processes compared with feeding sugar‐based solution (Lakshmanan et al., 2020). Specifically, based on stoichiometric estimations, 1 g of linoleic acid could yield 1.38 g of polyhydroxybutyrate (PHB), whereas glucose theoretically yields 0.48 g_PHB_ g_Glucose_^−1^ (Akiyama et al., 2003). In reality, PHA yields from plant oils are typically in the range of 0.6–0.8 g_PHA_ g_Oil_^−1^ (Gutschmann et al., 2019; Kahar et al., 2004; Ng et al., 2010; Riedel et al., 2012). While plant oils are excellent renewable carbon sources for PHA production, most of the plant oils are a food and farm‐grown, which begets a “plate vs plastic” controversy (Rosenboom et al., 2022). Moving away from first‐generation plant oils to oleaginous waste as feedstock not only curbs this debate, but also promotes the idea of a circular economy. Animal by‐ and coproducts, in particular waste animal fat (WAF), are available in large quantities and can be used for multiple higher value applications (Toldrá et al., 2021). In general, WAF contains a higher fraction of saturated fatty acids compared with (waste) plant oil, which is reflected by a higher melting temperature (*T*_m_). Consequently, the feedstock is solid at room temperature and difficult to consume by microorganisms, which makes the process development more challenging (Riedel et al., 2015). One strategy is the conversion of WAF to fatty acid methyl esters and raw glycerol via transesterification and the subsequent utilization of the proper fractions as substrate for PHA or biodiesel production (Muhr et al., 2013). In contrast, Riedel et al. reported the direct use of low‐quality WAF and succeeded to produce up to 27 g L^−1^ PHA (Riedel et al., 2015). Although WAF is a low‐cost feedstock contributing to reduction of PHA production costs (Koller et al., 2018), further cost reductions are required during the downstream processing of the polymer (Kosseva & Rusbandi, 2020).

*Ralstonia eutropha* (also known as *Cupriavidus necator*) is a bacterium that has been cultivated to produce PHA from various waste streams (Sohn et al., 2021). This bacterium has been used to produce PHA from plant‐based oleaginous waste streams such as waste frying oil and oil from coffee grounds (Brigham & Riedel, 2018). Additionally, animal‐based waste streams have been successfully applied to PHA production with *R. eutropha* (Kettl et al., 2012; Koller & Braunegg, 2015; Riedel et al., 2015; Rodríguez et al., 2021; Saad et al., 2021; Titz et al., 2012). Even though there are multiple advantages of using oleaginous feedstocks, it should be emphasized that these substrates increase the complexity of the bioprocess, as, due to their hydrophobicity, they do not dissolve in an aqueous growth medium. Emulsions must be formed to facilitate substrate availability, which is realized either by adding emulsifiers to the media (Budde et al., 2011a) or by taking advantage of the extracellular lipases of *R. eutropha*, which form natural emulsions that are potentially stabilized by extracellular polysaccharides (Gutschmann et al., 2021; Lu et al., 2013). While a wild‐type strain of *R. eutropha*, H16, contains a native PHA synthase (PhaC), which integrates short‐chain‐length monomers (*scl*, C‐atoms <6) into the polymer, an engineered strain of *R. eutropha*, Re2058/pCB113, expresses a heterologous PhaC enzyme that allows the synthesis of the copolymer poly(hydroxybutyrate‐*co*‐hydroxyhexanoate) (P [HB‐*co*‐HHx]) (Budde et al., 2011b). Such copolymers of *scl* and medium‐chain‐length (*mcl*, 6 ≥≤ C‐atoms ≤14) monomers possess superior properties regarding flexibility, ductility, crystallinity and toughness compared with *scl*‐homopolymers (Noda et al., 2005).

Another important aspect is that low‐cost PHA production is only feasible if it is carried out at sufficiently large scale (>100 m^3^). In contrast to laboratory‐scale processes, mass transfer issues due to limited power inputs are critical elements of large‐scale fermentations, which result in the formation of gradients of dissolved oxygen (DO), dissolved carbon dioxide, pH and substrate (Noorman et al., 2018). Such gradients can be mimicked with multiple scale‐down set‐ups already at laboratory‐scale and multiple process analytical technologies (PAT) tools, for example flow cytometry, are available to monitor the physiological cell response (Lemoine et al., 2017; Neubauer & Junne, 2016). A valuable PAT tool for PHA process development is *in‐line* photon density wave (PDW) spectroscopy, which allows real‐time quantitative monitoring of growth and PHA formation during *R. eutropha* plant oil cultivations (Gutschmann et al., 2019).

In this study, we introduce and evaluate a set‐up for applying a tailored feeding profile for solid WAF in cultivations with *R. eutropha* strain Re2058/pCB113 in laboratory‐ and pilot‐scale bioreactors. Flow cytometry and PDW spectroscopy are applied as PAT tools for assessing the process performance, including cell growth and PHA production.

## EXPERIMENTAL PROCEDURES

### Bacterial strain and growth media

Cultivations were performed with the engineered *R. eutropha* strain Re2058/pCB113 (Budde et al., 2011b), which produces the *scl‐mcl* copolymer P(HB‐*co*‐HHx) when grown on oleaginous substrates. Instead of the native PhaC, this strain harbours a PhaC from *Rhodococcus aetherivorans* and additionally an enoyl‐CoA hydratase (PhaJ) from *Pseudomonas aeruginosa*, which allows to specifically channel hexenoyl‐CoA from the β‐oxidation towards PHA synthesis (Budde et al., 2011b).

Tryptic soy broth (TSB) media, agar plates and mineral salt media (MSM) compositions have been described previously (Gutschmann et al., 2019). TSB agar plates and complex media additionally contained 200 μg ml^−1^ kanamycin sulfate for plasmid stability. In MSM, no addition of kanamycin was necessary, due to proline auxotrophy of the parental strain Re2058 and the presence of an intact proC gene on the pCB113 plasmid (Budde et al., 2011b). Initially, the MSM contained 10 g L^−1^ canola oil and 4.45 g L^−1^ urea as the main carbon and nitrogen sources. The amount of urea was chosen so that nitrogen is the limiting component in the medium and nitrogen depletion triggers maximum PHA formation. In total, 50 g L^−1^ WAF of porcine origin provided by ANiMOX GmbH was fed to the cultivations to provide further carbon to the system.

The physical and chemical characteristics of the WAF used for the cultivations in this study are shown in the Supporting Information in Table S1.

### Laboratory‐scale cultivation conditions

**FIGURE 1:**
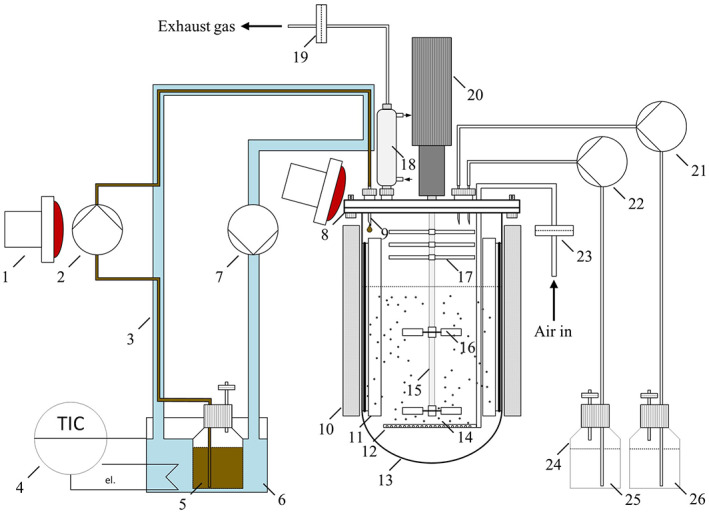
Schematic representation of the experimental set‐up for continuously feeding solid waste animal fat into a 6.7‐L laboratory‐scale bioreactor: (1) infrared lamp for heating; (2) peristaltic feeding pump; (3) double‐jacket tubing; (4) thermostat; (5) fat feeding bottle; (6) water bath for heating; (7) thermostat pump; (8) bioreactor lid: (9) needle; (10) heating blanket; (11) baffles; (12) ringsparger; (13) bioreactor vessel; (14) air bubble; (15) stirrer shaft; (16) Rushton turbine; (17) cable tie as mechanical foam breaker; (18) condenser; (19) exhaust gas filter; (20) motor; (21) base pump; (22) acid pump; (23) air inlet filter; (24) bottle for sterile substance addition; (25) acid for pH control; (26) base for pH control.

Initially, a cryoculture of *R. eutropha* Re2058/pCB113 was streaked on a TSB agar plate and incubated for 3–4 days at 30°C. A single colony was used to inoculate 10 ml TSB media (first preculture) in a 125‐ml Ultra Yield flask (Thomson Instrument Company) covered with an AirOtop enhanced flask seal (Thomson Instrument Company) for 14–16 h. At an OD_600_ ≥ 5, 3 ml of the first preculture were used to inoculate the second preculture (300 ml MSM). The second precultures were incubated in 1‐L DURAN baffled glass flasks with a GL45 thread (DWK Life Sciences GmbH) sealed with an AirOtop membrane. The first and second precultures were incubated at 30°C and shaken at 200 rpm (Kühner LT‐X incubator, Adolf Kühner AG, Switzerland, 25 mm amplitude). After 24 h of incubation, the second preculture was used to inoculate a 6.6‐L bioreactor (BIOSTAT Aplus, Sartorius AG) containing 2.7 L MSM. The bioreactor was equipped with two six‐blade Rushton impellers with a distance of 96 mm. The cultures were aerated with air using a mass flow controller (red‐y smart meter GSM, Vögtlin Instruments GmbH, Muttenz, Switzerland). Reference cultivations were constantly aerated with 1.5 L min^−1^ (0.5 vvm), whereas scale‐down cultivations were periodically aerated with 0.5 vvm for 12 min and subsequently the aeration was turned off for 3 min to achieve temporary DO depletions. This strategy was applied as a tool to mimic DO gradients, which occur at large scale. The DO levels of the reference cultivations were maintained above 40% by manually increasing the stirrer speed by 50 rpm whenever the DO dropped below 40%. The stirring speed of the scale‐down cultivations was adjusted simultaneously with the reference cultivations. The 50 g L^−1^ WAF were continuously fed with a constant feeding rate into the reactor from 10 h to 42 h. Due to the high T_m_ of the WAF, it was liquefied at 80°C and continuously heated in a double‐jacket tube to prevent clogging as schematically shown in Figure 1. The double‐jacket tubing system was created with two silicon tubes: the smaller tube used for pumping the WAF had an inner diameter of 2 mm and an outer diameter of 6 mm. To insert the smaller tube into a larger tube (inner diameter 12 mm, outer diameter 16 mm), a 6 mm pritchel was used to make four holes in the larger tube: one for entering the tube, two for the part of the thin tube inserted into the pump head and one for leaving the large tube to connect the bioreactor. Silicone caulk was used to seal the parts where the small tube was guided through the holes. Parts of the tube that were not covered by the double‐jacket tube (connection to the bioreactor and the part of tube inserted into the pump head) were maintained at 45°C using a temperature‐controlled power socket connected to infrared lamps.

### Pilot‐scale cultivation conditions

The seedtrain from the cryostock to the second preculture were identical to the laboratory‐scale cultivations. A third preculture in the laboratory‐scale bioreactor containing 4.75 L MSM was inoculated with 250 ml of the second preculture. The DO of the third preculture was maintained above 40% using a stirrer cascade between 200 and 950 rpm. The third preculture was aerated with 0.5 vvm and incubated for 18 h.

The pilot‐scale cultivations were carried out in a 150‐L bioreactor (P150, Bioengineering AG) equipped with three, 6‐blade Rushton impellers with a distance of 26 cm between them. The bioreactor had an initial working volume of 85 L (80 L MSM inoculated with 5 L of the third preculture). Initially, the culture was aerated with 0.5 vvm and mixed with an initial stirrer speed of 80 rpm. The stirrer speed and air supply were manually adjusted throughout the cultivations to prevent oxygen limitation: air supply was reduced stepwise as soon as foaming occurred and the stirrer speed was increased by 25 rpm whenever DO dropped below 40% up to a maximum of 750 rpm. The temperature was kept constant at 30°C and pH was kept at 6.8 ± 0.2 using 6 M NaOH and 3 M H_3_PO_4_. Antifoam agent (Nol‐LG126, Adeka) was added undiluted to the culture whenever heavy foaming occurred. In total of 200 ml antifoam agent was added. Liquefied WAF was added with the same set‐up shown in Figure 1, but a slower constant feeding was applied to keep a low WAF level in the reactor and thus reduce foaming: Feeding started after 11 h with 85 g h^−1^, it was increased to 127.5 g h^−1^ after 26 h and reduced to the initial feeding rate after 36 h until a total of 50 g L^−1^ WAF was added after 56 h. Determination of the volumetric oxygen transfer coefficient.

The (*k*_L_ *a*) was determined using the dynamic gassing out method. Prior to the experiments, the response time *τ* of the DO probes were determined to calculate the real DO values according to Equation 1.

(1) $${\mathrm{DO}}_{\text{real}}={\mathrm{DO}}_{\text{displayed}}\left(1+e^{\frac{-t}{\;\tau }}\right)$$

Experiments were carried out with a working volume of 3 L at a constant aeration rate of 0.5 vvm. The *k*_L_ *a* value was determined according to Equation 2 using the response time adapted DO values, where DO* denotes the maximum measured DO concentration under the applied conditions and DO_i_ is the measured concentration at timepoint *t*_i_.

(2) $$k_{L}a=\frac{\ln\left(\frac{{\mathrm{DO}}^{*}-{\mathrm{DO}}_{i}}{{\mathrm{DO}}^{*}-{\mathrm{DO}}_{i+1}}\right)}{t_{i+1}-t_{i}}$$

### Off‐line analytical methods

Aliquots of 5–15 ml were sampled in pre‐weighed 15‐ml polypropylene test tubes for the determination of the CDW and PHA content. The samples were centrifuged for 15 min at 6000 *g* and pellets were washed with a mixture of 5 ml cold deionized water (DI) and 2 ml cold hexane to remove the residual lipids. The washed pellets were resuspended in 2–4 ml cold DI water and dried by lyophilization.

The PHA content was determined using a gas chromatograph (GC) (GC‐2010 Plus, Shimadzu Corp.) equipped with a flame ionization detection (FID) and a DB‐WAX column (15 m × 0.32 mm × 0.5 μm, Agilent Technologies Inc.). The detailed method for quantification of the PHA and its monomeric composition was described previously (Bartels et al., 2020).

The RCDW was determined by subtraction the PHA concentration from the CDW concentration. The STY was obtained by dividing the PHA concentration by the process time.

Ammonia as a product from urea cleavage was quantified in the supernatants to determine the time point of nitrogen depletion in the cultures. Therefore, a pipetting robot with the NH_3_ Bio HT test kit (Cedex Bio HT Analyser, Roche Diagnostics International AG) was used.

### Photon density wave spectroscopy

A multifiber *in‐line* PDW spectroscopy probe was integrated into the bioreactor through a DN25 port as described previously (Gutschmann et al., 2019). This set‐up was used for a real‐time determination of the reduced scattering coefficient *μ*_s_' and the absorption coefficient *μ*_a_ at 638 nm with a temporal resolution of 2.5 min^−1^. Signal noise was reduced by a 10‐point moving average.

### Flow cytometry analysis

Aliquots of 1–2 ml were sampled in 2‐ml reaction tubes for flow cytometry analysis. The samples were harvested by centrifugation (6000 *g*, 21°C), residual oil/ fat was removed from the side of the reaction tubes with a cellulose cloth and the cells were subsequently resuspended in phosphate‐buffered saline (PBS; composition: 8.0 g L^−1^ NaCl, 0.2 g L^−1^ KCl, 1.44 g L^−1^ Na_2_HPO_4_ × 2H_2_O, 0.24 g L^−1^ KH_2_PO_4_, pH adjusted to 6.8 with 1 M NaOH). The washing procedure was repeated twice to remove all residual oil. Subsequently, the samples were diluted with PBS to a final concentration of 0.5–2 × 10^7^ particles ml^−1^ for flow cytometry analysis. Flow cytometry analysis was carried out using a MACSQuant Analyser 10 (Miltenyi Biotec). The instrument was calibrated with a commercially available calibration solution (MACSQuant Calibration Beads, Miltenyi Biotec). The instrument was operated with the following experiment settings: Low flow rate (25 μl min^−1^), gentle mixing before injection, standard injection speed, 80 μl uptake volume, acquisition of maximum 80,000 events.

For the quantification of propidium iodide (PI) stained cells, the particles were excited at 488 nm and fluorescens was detected in the range of 655–730 nm (channel B3). A design of experiment approach (Table S2) was conducted to establish a PI staining method for the strain varying the dye concentration, the staining time and the staining temperature. For the final procedure, a second round of experiments was carried out adapting the dye concentrations in a range of 0.25–15 μg ml^−1^. All incubations were carried out in the dark. The results of the titration experiments are shown in the Figure S1. The final procedure employed a staining time of 3 min, dye concentration of 6 μg ml^−1^ and was stained at room temperature. Cells were incubated at 80°C for 10–20 min prior the staining as a positive control and unstained cells were used as a negative control.

## RESULTS AND DISCUSSION

Prior to performing bioreactor cultivations, one key objective was to develop a new feeding system (Figure 1) to overcome many operational challenges faced when working with solid WAF as a feedstock (Riedel et al., 2015). The feeding system consisted of a double‐jacket silicone tubing system, which allowed to sufficiently heat the entire feed line to keep the WAF liquified. On one hand, this system allows for implementation of a slow but continuous WAF feed, resulting in a reduction of foaming in the culture, caused by an excess quantity of fatty acids that resulted from a pulse‐based feeding strategy in a previous study (Riedel et al., 2015). On the contrary, a slower feeding speed would increase the probability of WAF solidification in the tubing, which was prevented by sufficient heating over the entire feeding line. When scaling up the process further to industrial‐scale production, feeding tanks are usually used, which can be heated, and faster pumping rates are applied, which would avoid the solidification problem and simplify the process further. Tailored process equipment such as screw pumps could also be a solution to implement a WAF feeding at large scale. Consequently, the developed feeding set‐up is a useful tool for process development at laboratory‐ and pilot‐scale.

In this study, the feasibility of using the developed system is demonstrated for bioreactor cultivations at 6.6‐L laboratory‐ and 150‐L pilot‐scale. The laboratory‐scale cultivations were further evaluated by applying PDW spectroscopy as a real‐time monitoring tool and testing the robustness against oxygen gradients. An oscillating aeration was applied as a tool to mimic such gradients.

### Laboratory‐scale cultivations with constant aeration

**FIGURE 2:**
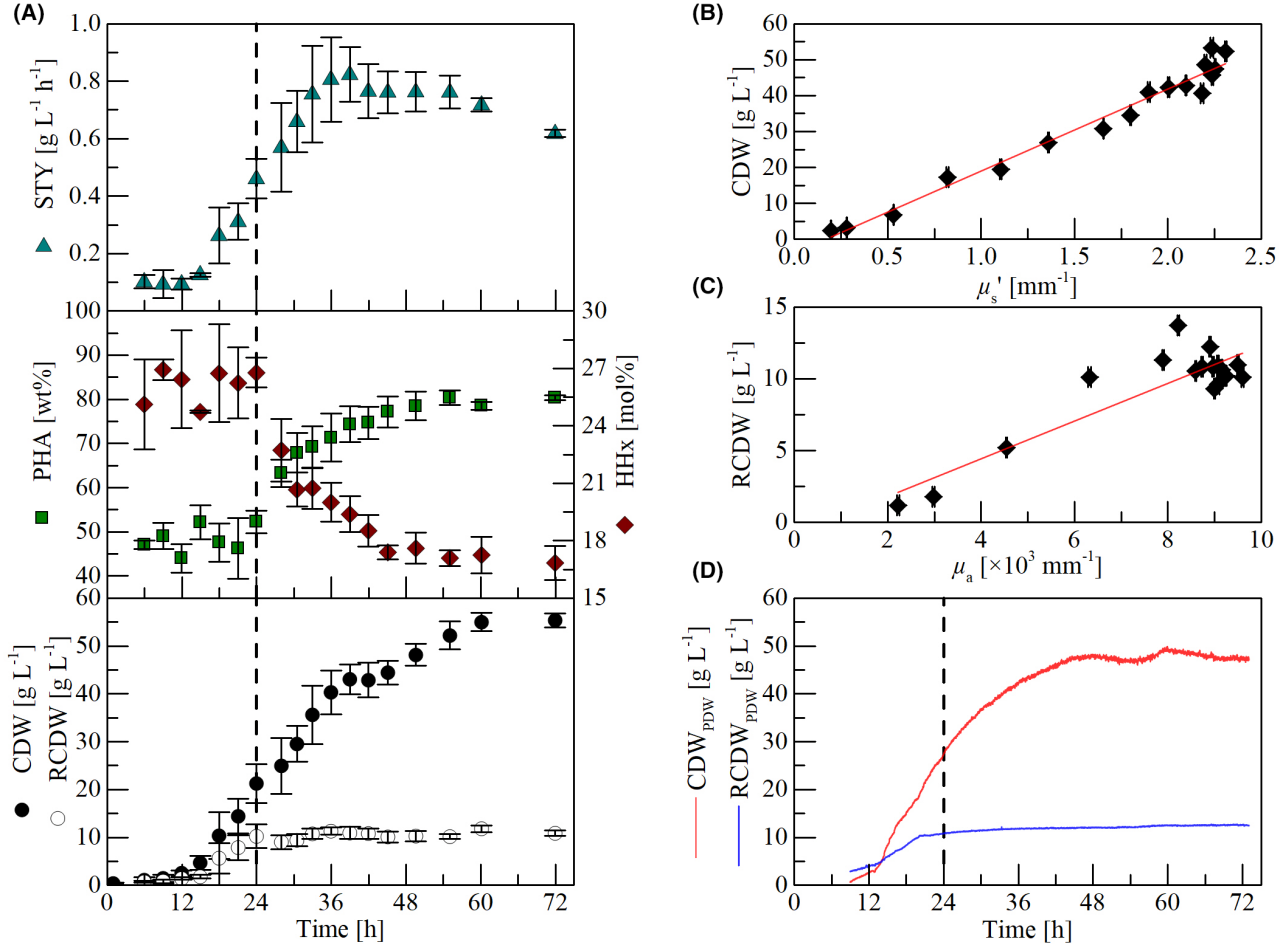
6.7‐L laboratory‐scale cultivations of *R. eutropha* Re2058/pCB113 for P(HB‐*co*‐HHx) production. The cultures initially contained 10 g L^−1^ canola oil as the main carbon source to form an initial emulsion and subsequently thermally liquefied waste animal fat was continuously fed from 10 h until 42 h to a total carbon concentration of 60 g L^−1^. The cultures were constantly aerated with 0.5 vvm air. The vertical dashed line represents the time point of nitrogen limitation. Results of the ammonia measurement are shown in Table S3. (A) *off‐line* results of three independent cultivations. STY = space time yield of P(HB‐*co*‐HHx) in g L^−1^ h^−1^, PHA = polyhydroxyalkanoate content of cell dry weight in wt%, HHx = HHx content of P(HB‐*co*‐HHx) in mol%, CDW = cell dry weight in g L^−1^, RCDW = residual cell dry weight in g L^−1^.Error bars indicate standard deviation of the three cultivations. (B) correlation of CDW with the scattering coefficient *μ*_s_' signal of the PDW spectroscopy (*R* ^2^ = 0.987). (C) correlation of RCDW with the absorption coefficient *μ*_a_ signal of the PDW spectroscopy (*R* ^2^ = 0.891). (D) real‐time CDW and RCDW values calculated from the *in*‐*line* PDW spectroscopy signals.

After 55 h, the cultivations yielded on average 52 g L^−1^ cell dry weight (CDW) and 42 g L^−1^ PHA, which corresponds to a space time yield (STY) of 0.76 g_PHA_ L^−1^ h^−1^ (Figure 2A). The CDW increases slightly further to 55 g L^−1^ containing 80 wt% PHA with an HHx content of 17 mol% after 72 h, which corresponds to a STY of 0.62 g_PHA_ L^−1^ h^−1^ (Figure 2A). In cultivations with the same strain and same nitrogen content, a 42% lower STY was obtained (Riedel et al., 2015). This drastically lower yield results from a relatively low PHA content of 58 wt% in the former study, which might be the consequence of the applied pulse feeding. Such a feeding results in heavy foaming, which might lead to substrate loss at reactor walls or cells were damaged due to a high content of free fatty acids (Riedel et al., 2015). A reduction in the HHx content was observed after the onset of nitrogen depletion (Figure 2A), which was also reported for growth of the same strain on palm oil (Budde et al., 2011b; Riedel et al., 2012). This phenomenon can be explained by an increased availability of acetyl‐CoA for PHA synthesis caused by a shutdown of growth‐related metabolism (TCA, anabolism) after nitrogen gets depleted, which gradually increases the molar HB fraction in the polymer. Higher PHA titers than those in this study were achieved in cultivations with *R. eutropha* to cell densities >100 g L^−1^ in similar cultivation times for multiple other renewable feedstocks (Arikawa et al., 2017; Arikawa & Matsumoto, 2016; Riedel et al., 2012; Santolin et al., 2021; Sato et al., 2015). Such results are likely to be achieved with WAF as a feedstock by extending the growth phase through the addition of additional nitrogen, but the scope of this study was to establish a possibility to fine‐tune the solid WAF feeding profile.

Application of *in‐line* PDW spectroscopy as a process monitoring tool allowed a correlation of the CDW with the scattering signal *μ*_s_' and of the residual cell dry weight (RCDW) with the absorption signal *μ*_a_ (Figure 2B, C). These correlations facilitate a real‐time estimation of the CDW and RCDW during the process (Figure 2D). Absorption and scattering increased during the growth phase, but absorption stayed constant after nitrogen depletion, which means that the PHA granules only contributed to the scattering but not the absorption signal. The same effect has been reported in a previous study where wild‐type *R. eutropha* strain H16 was grown on virgin plant oil (Gutschmann et al., 2019). It is very likely that the same monitoring strategy could be applied to other PHA‐producing organisms. Using this PAT tool to monitor such important process parameters independently of the feedstock and organism allows the development of a flexible process control strategy, which is highly desired when working with process and raw material variations (Eifert et al., 2020).

### Laboratory‐scale cultivations with oscillating aeration

**FIGURE 3:**
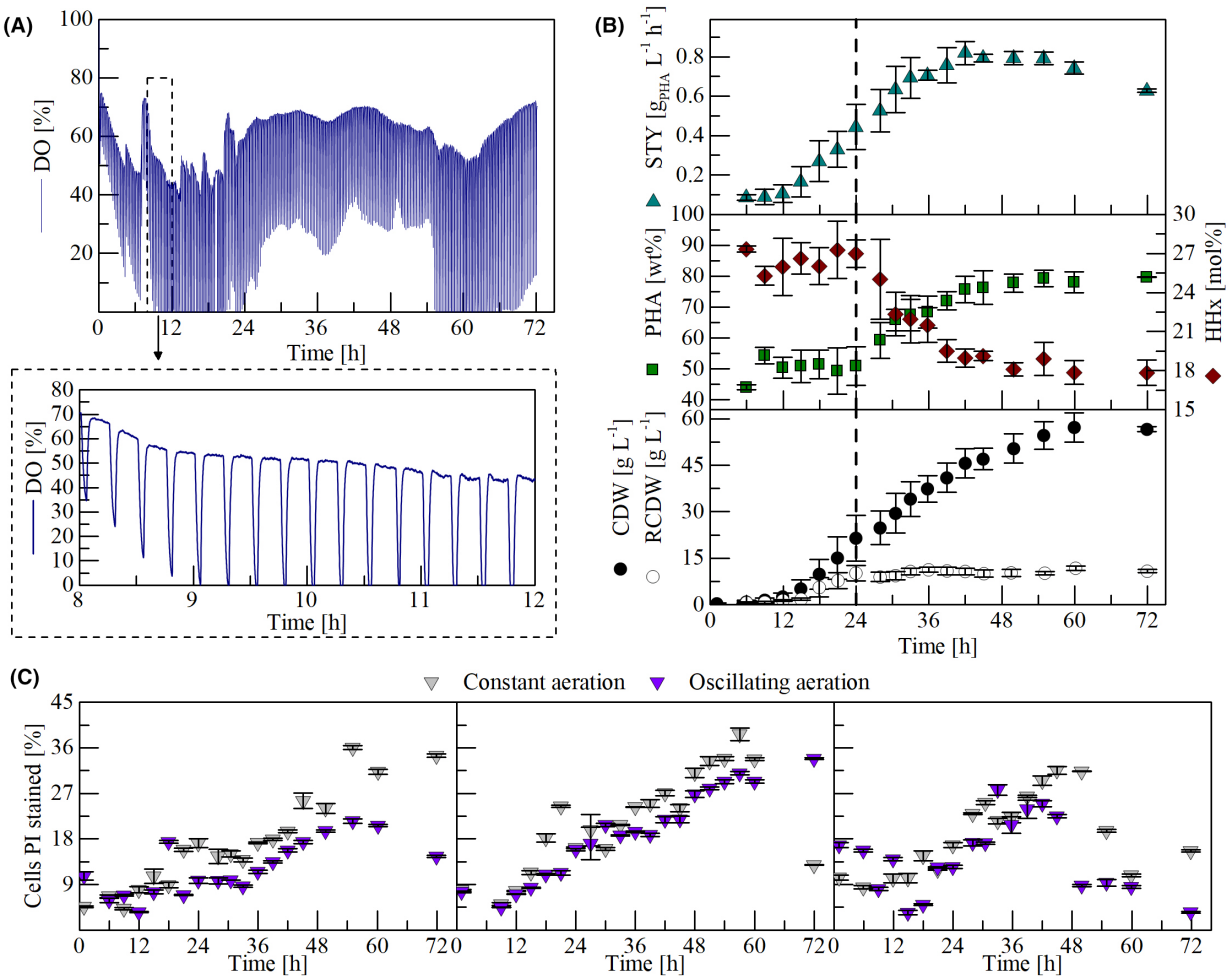
6.7‐L laboratory‐scale cultivations of *R. eutropha* Re2058/pCB113 for P(HB‐*co*‐HHx) production with oscillating aeration. (A) Exemplary dissolved oxygen (DO) profile of one cultivation with a zoom (indicated by dashed line) of the data between 8 and 12 h. (B) *off‐line* results with error bars indicating the standard deviation of three independent cultivations. The vertical dashed line represents the time point of nitrogen limitation. Results of the ammonia measurement are shown in Table S3. STY = space time yield of P(HB‐*co*‐HHx) in g L^−1^ h^−1^, PHA = polyhydroxyalkanoate content of cell dry weight in wt%, HHx = HHx content of P(HB‐*co*‐HHx) in mol%, CDW = cell dry weight in g L^−1^, RCDW = residual cell dry weight in g L^−1^. (C) Comparison of propidium iodide (PI) stained cells from 3 × 2 parallel running bioreactor cultivations, inoculated from the same preculture but aerated with different strategies: Constant (see *off‐line* data in Figure 2A) or oscillating aeration (see *off‐line* data in B). Error bars indicate standard deviation of three technical staining replicates.

Aerobic fermentation processes are most often limited by mass transfer at large scale, which results in longer mixing times. This results in the formation of gradients in the fed substrates and oxygen supply, especially at higher cell densities when the local consumption rates exceed the mass transfer rates. This phenomenon is usually not present in well‐mixed laboratory‐scale cultivations but can be simulated with different approaches (Neubauer & Junne, 2016). To further evaluate the robustness of the developed WAF process (Figure 2), whether the availability of oxygen is a major parameter for an effective PHA production process, a pulse‐based aeration scenario was tested. Cultures were aerated for 12 min, and subsequently, the aeration was turned off for 3 min. While the DO was kept >40% in the reference cultivations, the oscillating aeration led to periods of oxygen depletion, especially during the growth phase (Figure 3A). Surprisingly, with such constraints, the cultivations yielded in average 57 g L^−1^ CDW containing 80 wt% PHA with an HHx content of 18 mol% and a resulting STY of 0.63 g_PHA_ L^−1^ h^−1^ after 72 h (Figure 3B), which is a slightly higher CDW compared to the cultivations with a constant aeration (Figure 2). Although this difference is not significant, it demonstrates the robustness of the *R. eutropha* cells and the developed process to the applied DO gradients. Nevertheless, it is likely that extended periods of DO depletion may show an impact on the process performance as physiological responses are very likely due to the versatile physiology of *R*. *eutropha* which can adapt to anaerobic conditions (Pohlmann et al., 2006).

For a more detailed understanding of the impact on the cell physiology, cell viability was assessed by flow cytometry with propidium iodide (PI) staining. The dye can only enter cells with damaged membrane integrity (i.e., non‐viable cells) where it binds to negatively charged DNA, leading to a fluorescence signal (Al‐Rubeai et al., 1997). An increasing proportion of PI‐stained cells was detected in all fermentations independent of the aeration strategy, which especially increased after nitrogen limitation (Figure 3C). More than two‐thirds of the cells were not stained with PI at the end of the cultivations, which means they were still viable at that point, which is likely to be caused by PHA granules enhancing the bacterial robustness (Obruca et al., 2020). Interestingly, in all parallel experiments, the proportion of PI‐stained cells was a little higher in fermentations with constant aeration. It is very likely that the constant aeration led to more oxidative damage, which typically occurs during aerobic fermentations (Chiang & Schellhorn, 2012; StJohn et al., 2001). Recently, it was shown that *R*. *eutropha* takes up antioxidants from palm oil in aerobic fermentations (Gutschmann et al., 2021), which might not be present in WAF and consequently cells are more easily damaged because of the decreased antioxidant availability.

### Determination of the volumetric transfer coefficient

The influences of oleaginous substrates on the volumetric oxygen transfer coefficient (*k*_L_ *a*) were determined in the used bioreactor systems under the applied cultivation conditions. Canola oil was used as representative for oleaginous substrates, as WAF could not be used for these experiments, due to its high melting temperature. The maximal *k*_L_ *a* decreased from 170 to 120 h^−1^ (Figure S2) when canola oil was added to the media. A reduced *k*_L_ *a* due to plant oil in the media was also described for palm oil containing media (Sauid & Murthy, 2010). A corresponding maximal oxygen transfer rate (OTR) of 44 mmol L^−1^ h^−1^ is low compared to possible transfer rates of up 500–700 mmol L^−1^ h^−1^ at industrial‐scale (Noorman et al., 2018). Consequently, the here shown WAF‐based process can be operated at low power inputs, which makes the process more economical. Additionally, applying the used slow feeding is beneficial for the OTR by keeping the oleaginous substrate at low levels and reduces the foaming.

### Pilot‐scale cultivation

**FIGURE 4:**
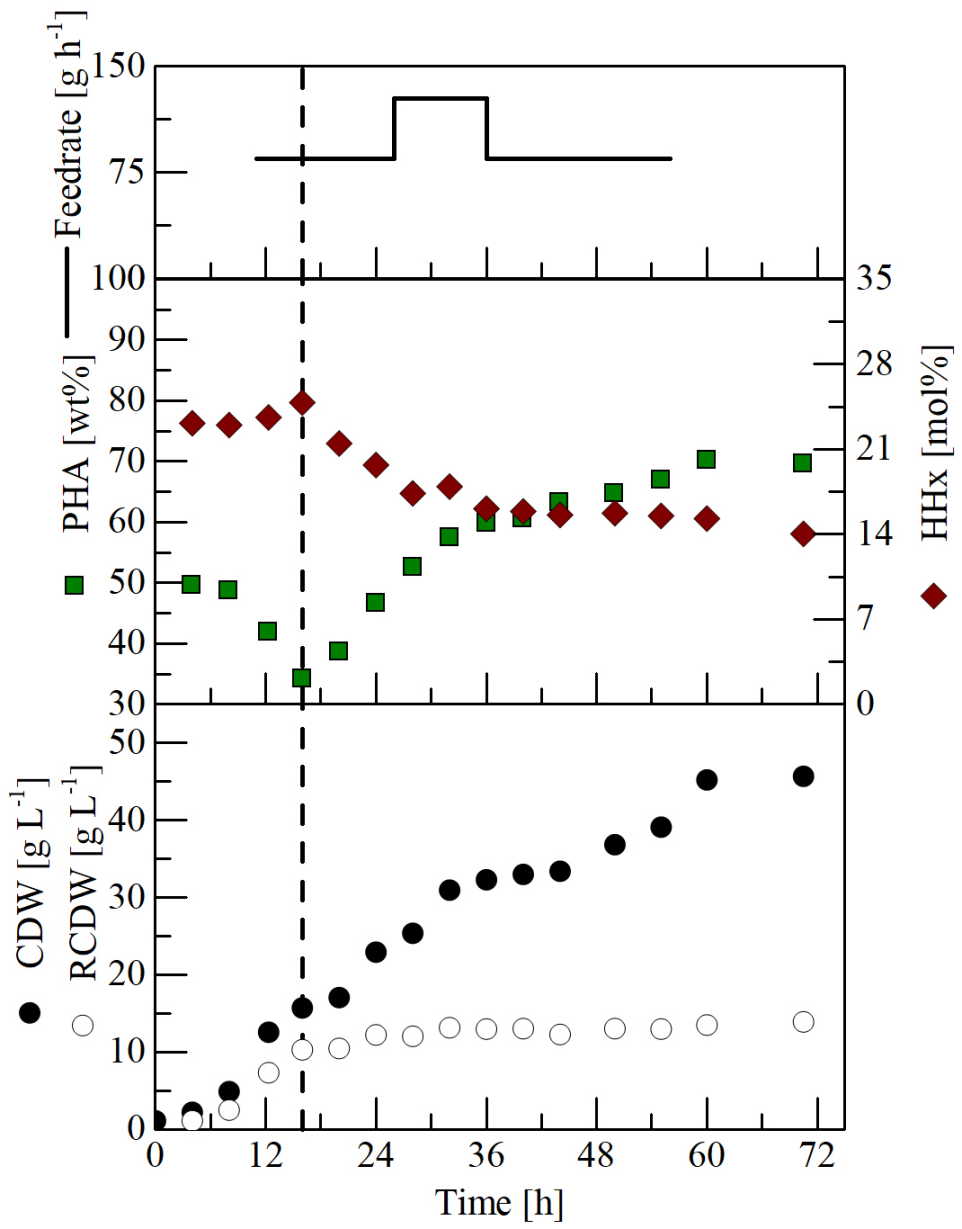
150‐L pilot‐scale cultivation of *R. eutropha* Re2058/pCB113 for P(HB‐*co*‐HHx) production. The culture initially contained 10 g L^−1^ canola oil and 4.5 g L^−1^ urea in 85 L mineral salt medium. From 10 h to 56 h solid waste animal fat (WAF) was fed to a total concentration of 50 g L^−1^ by continuously pumping liquefied WAF through a double‐jacket tubing system. Vertical dashed lines indicate the time point of nitrogen depletion. CDW, cell dry weight in g L^−1^; HHx = HHx content of P(HB‐*co*‐HHx) in mol%; PHA = polyhydroxyalkanoate content of cell dry weight in wt%; RCDW = residual cell dry weight in g L^−1^.

After successfully applying the double‐jacket tubing system for feeding solid WAF at laboratory‐scale, the feeding system was used in a 150‐L pilot‐scale cultivation. In comparison with the laboratory‐scale cultivations, nitrogen was already depleted after 14 h and similar RCDW values to laboratory‐scale experiments were reached in that period (Figure 4). The seedtrain extension with a third preculture in a bioreactor most probably resulted in a more vital inoculum, which is better adapted to the hydrophobic substrate. Such an adaptation could be very beneficial with regards to faster substrate emulsification and consequently a better process performance. A similar effect was observed for lipase overexpression strains, which emulsified plant oil more rapidly than the *R. eutropha* wild‐type strain and subsequently had a reduced lag‐phase (Lu et al., 2013). Even though quick growth was observed after inoculation, a high PHA content (50 wt%) indicates that preculture was already under nitrogen limiting conditions and suggests that the incubation period of the third preculture could be shortened.

Compared with the laboratory‐scale cultivations, the cells accumulated only 70 wt% PHA, which resulted in a lower CDW and PHA concentration (Figure 4). The maximal CDW of 45 g L^−1^ was already achieved after 60 h, shortly after the substrate feeding was stopped. One possibility for the lower PHA accumulation is that not enough WAF was added to the cultivation due to a potential loss when changing feeding bottles, but scale‐up‐related effects (Noorman, 2011; Takors, 2012) may also have led to the slight decrease in PHA accumulation compared with the laboratory‐scale cultivations in this study and other studies described in the literature for this strain (Purama et al., 2018; Riedel et al., 2012; Saad et al., 2021; Thinagaran & Sudesh, 2017). Nevertheless, a high STY of 0.53 g_PHA_ L^−1^ h^−1^ at 60 h is a good rate to facilitate a low‐cost PHA production.

During operation of the reactor, foam could not be destroyed mechanically as in the laboratory‐scale cultivations, so an antifoam agent had to be used to minimize foaming, the aeration was reduced and the feeding rate was decreased when heavy foaming was observed at 36 h. Typically, application of antifoam agents increases process costs and interferes with downstream processing (Routledge, 2012), which is why different solutions need to be considered. Installation of a mechanical foam breaking system, such as a foam centrifuge, might allow cultivation to higher cell densities and should avoid the use of antifoam agents.

### PHA production from different animal waste streams

Slaughtering and processing of meat generates large quantities of by‐products, which are potential raw materials for multiple purposes (Toldrá et al., 2021). While the potential for conversion of animal waste streams to PHA with *R. eutropha* is known, only few studies focussed on that topic (Table 1). So far, only one wild‐type strain (H16) (Riedel et al., 2015; Taniguchi et al., 2003), a mutated wild‐type strain (H1 G^+^3) (Koller & Braunegg, 2015; Rodríguez et al., 2021) and two engineered strains (Re2058/pCB113, JR11) (Riedel et al., 2015; Rodríguez et al., 2021; Saad et al., 2021) have been used to convert animal waste streams to PHA. The only strain, which was engineered to enhance the PHA production from oleaginous animal waste is JR11, which expresses two heterologous extracellular lipases (Rodríguez et al., 2021). A similar strategy was also conducted in another study, where overexpression of an extracellular lipase enhanced the emulsification of palm oil and consequently reduced the overall process time (Lu et al., 2013). Such approaches demonstrate how strain engineering can contribute to a bioprocess optimization, but it is also important to develop efficient cultivation strategies for an improved product output. While shake flask cultivations in general have low yields and are not suitable for a commercial production, they are well suited for screening purposes and allow an initial evaluation of different animal by‐products (Table 1). Yields significantly improve in bioreactor studies, but operational challenges increase, which is probably why previous studies applied a pulse‐based feeding strategy (Koller & Braunegg, 2015; Riedel et al., 2015). In contrast, the current set‐up is rather simple to implement and allows for employment of tailored feeding profiles, such as a slow continuous feeding. In this study, the laboratory‐scale cultivations showed the highest CDW and PHA yields among published results, only a higher STY was reported for using saturated fatty acid methyl esters from slaughterhouse waste (Koller & Braunegg, 2015). The obtained PHA concentration at laboratory‐scale is 70% higher compared to results achieved with the same strain fed with a waste pork/cattle fat mixture (Riedel et al., 2015).

**TABLE 1:** Cultivation results of *R. eutropha* on different animal waste streams. CDW = cell dry weight, PHA = polyhydroxyalkanoate, STY = space time yield.

Carbon source	*R. eutropha* strain	Scale	Feeding strategy	CDW [g L^−1^]	PHA [wt%]	STY [g_PHA_ L^−1^ h^−1^]	References
Waste pork fat	Re2058/pCB113	Lab‐scale	Continuous	57.0	80	0.63	This study
Waste pork fat	Re2058/pCB113	Pilot scale	Continuous	45.0	70	0.53	This study
Waste pork/cattle fat mixture	Re2058/pCB113	Lab‐scale	Pulse‐based	45.0	58	0.36	Riedel et al. (2015)
Tallow	Re2058/pCB113	Lab‐scale	Pulse‐based	38.6	63	0.34	Riedel et al. (2015)
SFAE from hydrolyzed slaughterhouse waste	H1 G^+^3	Lab‐scale	Pulse‐based	34.9	80	0.94	Koller and Braunegg (2015)
Waste pork fat‐protein emulsion	Re2058/pCB113	Shake flask	‐	7.5	69	0.07	Saad et al. (2021)
Tallow	H16	Shake flask	‐	7.3	80	0.08	Taniguchi et al. (2003)
Waste chicken fat	Re2058/pCB113	Shake flask	‐	4.6	72	0.05	Riedel et al. (2015)
Waste swine/cattle fat mixture	H16	Shake flask	‐	4.5	72	0.04	Riedel et al. (2015)
Porcine jowl	JR11	Shake flask	‐	4.4	66	0.03	Rodríguez et al. (2021)
Waste pork fat greaves	Re2058/pCB113	Shake flask	‐	4.2	64	0.04	Saad et al. (2021)
Waste fish fat	Re2058/pCB113	Shake flask	‐	4.0	90	0.05	Saad et al. (2021)
Waste pork fat	H16	Shake flask	‐	4.0	73	0.04	Riedel et al. (2015)
Porcine membrane caul	JR11	Shake flask	‐	2.8	29	0.01	Rodríguez et al. (2021)
Waste pork fat	Re2058/pCB113	Shake flask	‐	2.5	76	0.03	Saad et al. (2021)
Tallow	H16	Shake flask	‐	2.5	61	0.02	Riedel et al. (2015)
Bovine udder	JR11	Shake flask	‐	2.2	18	0.004	Rodríguez et al. (2021)

Abbreviation: SFAE, saturated fatty acid methyl esters.

## CONCLUSIONS

A strategy for continuous feeding solid waste animal fat was developed to produce PHA at laboratory‐ and pilot‐scale yielding 45.6 and 31.5 g L^−1^ PHA, respectively. PDW spectroscopy allowed real‐time monitoring of growth and PHA production phases. Scale‐down cultivations with short periods of oxygen limitations during the growth phase had no negative impact on the process performance, which shows the robustness of *R. eutropha* and its suitability for large‐scale PHA production. Solid WAF could be applied as a cheap carbon source and we are confident that even higher cell densities and space–time yields can be achieved with such a feedstock.

## AUTHOR CONTRIBUTIONS

SLR and BG contributed to the conception and design of the study. BG, MMS, TS, ESS and AB carried out the experiments and analysis of the data. BG prepared the first draft of the manuscript. SLR, MM and PN were responsible for the project administration and funding acquisition. All authors contributed to the manuscript revision, read, and approved the submitted version.

## CONFLICT OF INTEREST

The authors declare no conflict of interests.

## Supporting information


Appendix S1

## Data Availability

The raw data supporting the conclusions of this article will be made available by the authors, without undue reservation.
