# Supplementary material for: Continuous feeding strategy for polyhydroxyalkanoate production from solid waste animal fat at laboratory‐ and pilot‐scale
Source: Microb Biotechnol. 2022 Aug 3;16(2):295–306. doi: 10.1111/1751-7915.14104 (PMC9871520; doi:10.1111/1751-7915.14104)
Supplement: Supplementary file 1 — Appendix S1 [file MBT2-16-295-s001.docx]

Supporting Information

Research Article

Continuous feeding strategy for polyhydroxyalkanoate production from solid waste animal fat at lab- and pilot-scale

Björn Gutschmann^1^, Matilde Maldonado Simões^1^, Thomas Schiewe^2^, Edith S. Schröter^1^, Marvin Münzberg^2^, Peter Neubauer^1^, Anika Bockisch^1,3^, Sebastian L. Riedel^1,*^

^1^ Technische Universität Berlin, Chair of Bioprocess Engineering, Berlin, Germany

^2^ innoFSPEC, University of Potsdam, Potsdam, Germany

^3^ Bio-PAT e.V., Berlin, Germany

* Correspondence: riedel@tu-berlin.de

**Table S1.** Characteristics of the waste animal fat used in this study. FFA = free fatty acids; N = nitrogen, T_m_ = melting temperature

| Origin | Dry matter [wt%] | Total fat [wt%] | FFA [%] | Total N [wt%] | Ash [wt%] | T_m_ [°C] | Main fatty acids [%] | | |
| --- | --- | --- | --- | --- | --- | --- | --- | --- | --- |
|  |  |  |  |  |  |  | C18:1-3 | C18:0 | C16:0 |
| Porcine | 99.2 | 98.4 | 0.4 | <0.02 | <0.04 | 48 | 53 | 18 | 24 |

**Table S2.** Design of experiment to study the influence of the propidium iodide (PI) concentration, temperature and incubation time on the staining efficiency and selectivity.

| ID # | PI Concentration [µg µL^–1^] | Staining time [min] | Temperature [°C] |
| --- | --- | --- | --- |
| 1 | 1 | 3 | 4 |
| 2 | 1 | 3 | 22 |
| 3 | 1 | 7 | 4 |
| 4 | 1 | 7 | 22 |
| 5 | 1 | 10 | 4 |
| 6 | 1 | 10 | 22 |
| 7 | 6.4 | 3 | 4 |
| 8 | 6.4 | 3 | 22 |
| 9 | 6.4 | 7 | 4 |
| 10 | 6.4 | 7 | 22 |
| 11 | 6.4 | 10 | 4 |
| 12 | 6.4 | 10 | 22 |
| 13 | 11.4 | 3 | 4 |
| 14 | 11.4 | 3 | 22 |
| 15 | 11.4 | 7 | 4 |
| 16 | 11.4 | 7 | 22 |
| 17 | 11.4 | 10 | 4 |
| 18 | 11.4 | 10 | 22 |


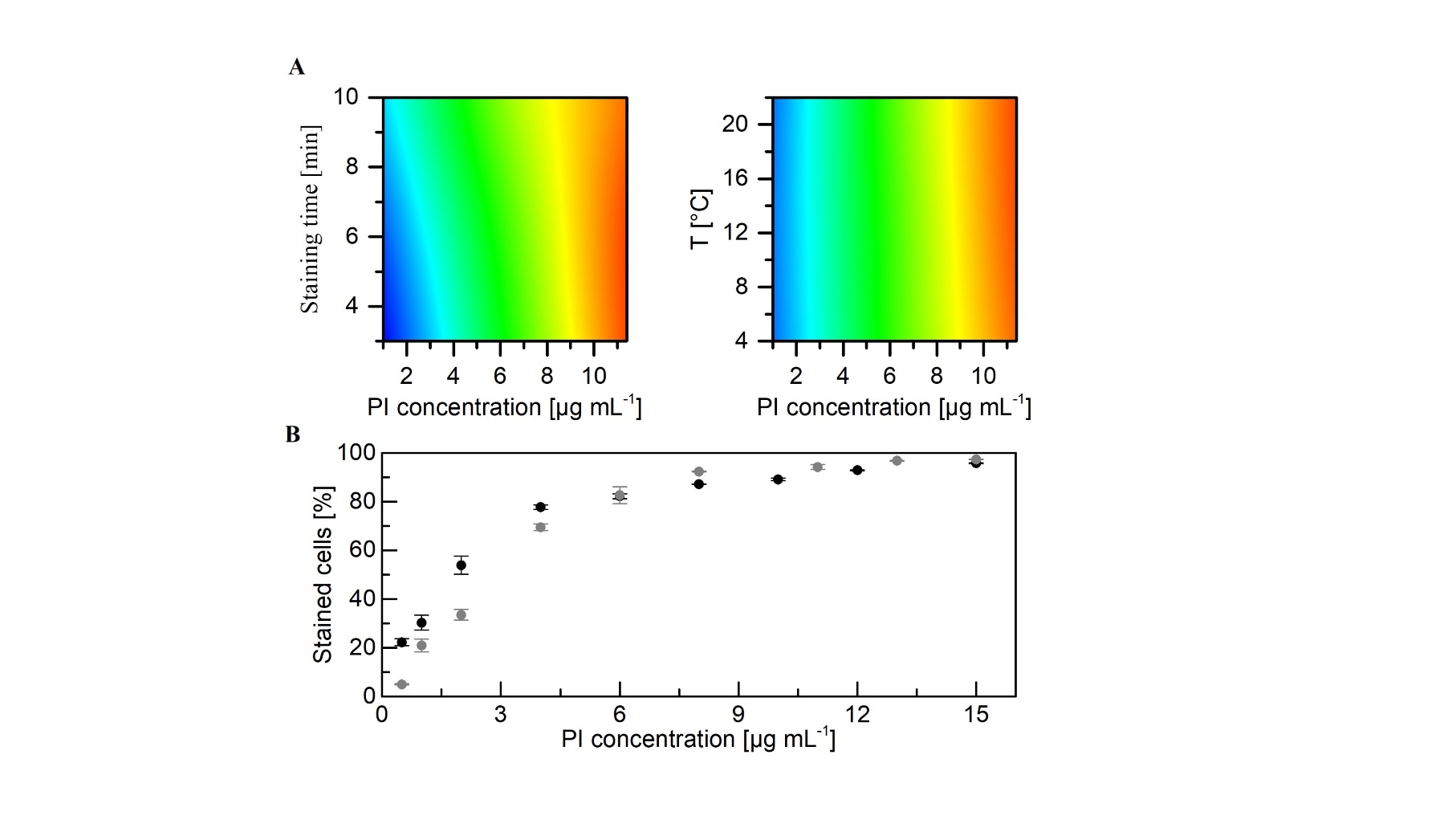


Figure S1: Determination of the propidium iodide (PI) staining procedure. **A** Variation of temperature, staining time and PI concentration as a first round of screening, approached with a design of experiment as shown in Table S1. Red represents the highest amount of stained cells and blue represents the lowest amount of stained cells. **B** Variation of the PI concentration at different staining times (grey: 3 min, black: 7 min) with cells stressed by incubation for 10 min at 80°C prior staining.

*
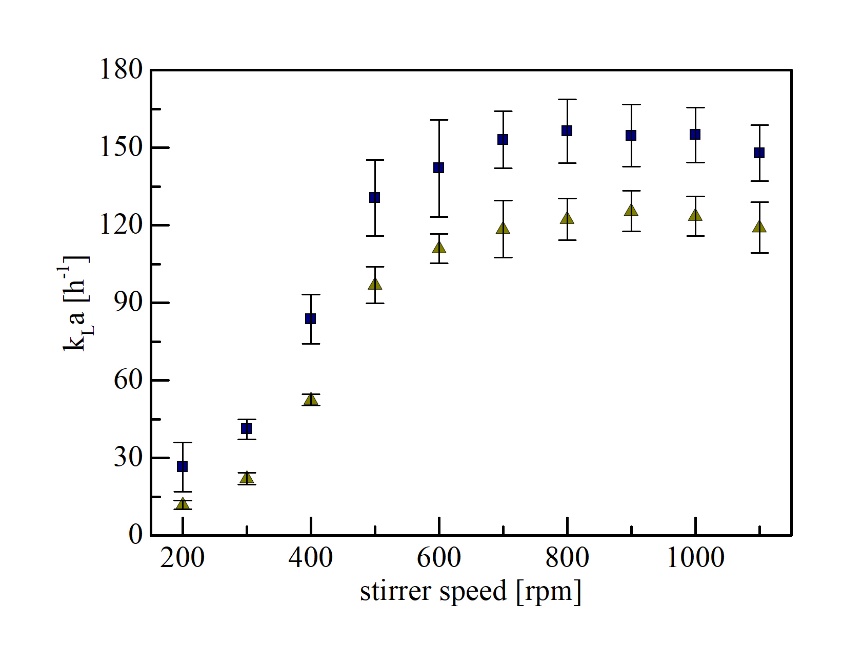
*

Figure S2: *k*_L_*a* values determined by the dynamic gassing out method in 3 L MSM without (blue squares) and with 10 g L^-1^ canola oil (yellow triangles) at different stirring rates aerated with 0.5 vvm air. Error bars represent minimum and maximum values of measurements with two amperometric dissolved oxygen probes.

**Table S3.** Ammonia concentrations determined during the cultivations.

| Time [h] | Ammonia content [mM] | |
| --- | --- | --- |
|  | Constant aeration | Oscillating aeration |
| 1 | 7.3 ± 0.2 | 6.9 ± 0.4 |
| 6 | 6.2 ± 0.5 | 6.3 ± 0.3 |
| 9 | 6.1 ± 0.1 | 6.0 ± 0.4 |
| 12 | 4.1 ± 0.5 | 4.3 ± 0.7 |
| 15 | 3.5 ± 0.3 | 3.3 ± 0.6 |
| 18 | 4.0 ± 0.3 | 5.0 ± 0.7 |
| 21 | 3.9 ± 0.4 | 6.9 ± 1.2 |
| 24 | <0.278* | <0.278*. |
| 28 | <0.278* | <0.278*. |
| 31 | <0.278* | <0.278*. |
| 33 | <0.278* | <0.278*. |
| 36 | <0.278* | <0.278* |
| 39 | <0.278* | <0.278* |
| 42 | <0.278*. | <0.278* |
| 45 | <0.278*. | <0.278*. |
| 50 | <0.278* | <0.278* |
| 55 | <0.278* | <0.278* |
| 60 | <0.278* | <0.278* |
| 72 | <0.278* | <0.278*. |
| * = below test range | | |
